# A Deep Learning-Based Algorithm for Identifying Precipitation Clouds Using Fengyun-4A Satellite Observation Data

**DOI:** 10.3390/s23156832

**Published:** 2023-07-31

**Authors:** Guangyi Ma, Jie Huang, Yonghong Zhang, Linglong Zhu, Kenny Thiam Choy Lim Kam Sian, Yixin Feng, Tianming Yu

**Affiliations:** 1 School of Electronics and Information Engineering, Nanjing University of Information Science and Technology, Nanjing 210044, China; gyma@nuist.edu.cn; 2 School of Automation, Nanjing University of Information Science and Technology, Nanjing 210044, China; 18168071335@163.com; 3 School of Internet of Things Engineering, Wuxi University, Wuxi 214105, China; llzhu@cwxu.edu.cn; 4 School of Atmospheric Science and Remote Sensing, Wuxi University, Wuxi 214105, China; kennylimks@cwxu.edu.cn; 5 Anhui Meteorological Information Center, Hefei 230031, China; 18752012969@163.com; 6 Tiantai Meteorological Bureau, Taizhou 317200, China; tianming9313@163.com

**Keywords:** precipitation cloud identification, Fengyun-4A, nychthemeron, deep learning

## Abstract

Rapid and accurate identification of precipitation clouds from satellite observations is essential for the research of quantitative precipitation estimation and precipitation nowcasting. In this study, we proposed a novel Convolutional Neural Network (CNN)-based algorithm for precipitation cloud identification (PCINet) in the daytime, nighttime, and nychthemeron. High spatiotemporal and multi-spectral information from the Fengyun-4A (FY-4A) satellite is utilized as the inputs, and a multi-scale structure and skip connection constraint strategy are presented in the framework of the algorithm to improve the precipitation cloud identification. Moreover, the effectiveness of visible/near-infrared spectral information in improving daytime precipitation cloud identification is explored. To evaluate this algorithm, we compare it with five other deep learning models used for image segmentation and perform qualitative and quantitative analyses of long-time series using data from 2021. In addition, two heavy precipitation events are selected to analyze the spatial distribution of precipitation cloud identification. Statistics and visualization of the experiment results show that the proposed model outperforms the baseline models in this task, and adding visible/near-infrared spectral information in the daytime can effectively improve model performance. More importantly, the proposed model can provide accurate and near-real-time results, which has important application in observing precipitation clouds.

## 1. Introduction

Precipitation estimation based on geostationary-Earth-orbiting (GEO) weather satellites has been widely used as critical information for meteorological and hydrological research such as drought modeling, soil moisture monitoring, and severe weather early warning [1,2]. Moreover, the GEO satellites can provide multi-spectral information with high spatial and temporal resolution, which is vital for precipitation monitoring. Its continuous observations of clouds and precipitation are highly beneficial for tracking and monitoring the changes in extreme weather systems [3,4]. Precipitation estimation algorithms based on GEO satellites mainly use Infrared (IR) data to establish an indirect relationship between the cloud top brightness and surface precipitation [5]. The premise of establishing this relationship is to accurately identify the precipitation clouds, which eliminates the interference of non-precipitation areas for further estimation of precipitation. So, the research on the identification of precipitation clouds is useful for improving precipitation estimation [6]. The rapid developments in satellite technology and sensors can provide multi-spectral observation data with higher spatiotemporal resolution. Coupled with advances in deep learning techniques and computing capabilities, this offers tremendous opportunities for developing an accurate model to identify precipitation clouds [7].

Multi-spectral observation data from satellites can approximately reflect the vertical structure of precipitation clouds, which provides a lot of information about the physical processes within the cloud [8]. For example, the IR sensors onboard GEO satellites can detect cloud top surface information, which is an approximate representation of cloud top temperature. In contrast, visible and near-infrared channels can be approximated for inversion of cloud properties such as particle size and optical thickness [9]. So, the fusion of information from multiple channels can provide richer physical information for the identification of precipitation clouds. For example, considering only the physical characteristics of clouds, the occurrence of convective precipitation is usually manifested in areas with lower cloud top temperature (CTT), higher cloud top height (CTH), and larger cloud depth. According to the research of [10,11], the brightness temperature in the 10.8 μm bandwidth can be used as a good proxy for the cloud top temperature. And the channel difference ΔT_6.25–10.7_ is calculated as the indicator of cloud top height. Kühnlein et al. [12] and Thies et al. [13] believe that the cloud phase (CP) has a great influence on the rainfall probabilities and intensities for convective precipitation events. And the research of [14,15] indicates that the channel differences between 10.7 μm and 13.5 μm can be used as information about the cloud phase. The water vapor (WV) band is critical for deriving precipitation, and the combination with CTT can improve the ability of precipitation cloud identification [16,17,18]. Lensky et al. [19] and Nauss et al. [20,21] have found that cloud areas with a high cloud water path (CWP) possess a higher rainfall probability and rate. In addition, the visible channel is a non-water absorption channel, and its radiation signal reflects the information on cloud thickness and cloud droplet particle density. The thicker the cloud, the more likely it is to produce precipitation. So, the visible channel signal is also an important indication of precipitation cloud identification. The near-infrared water absorption band signal is related to the cloud top size, and the particle size also contains precipitation information [22]. The visible and near-infrared channels can reflect the unique color and brightness of the precipitation cloud, but it is only valid in the daytime.

At present, researchers have achieved certain results in studying strong convective cloud cluster identification using satellite remote sensing technology. For different study regions, scholars have proposed the identification of strong convective cloud masses based on different brightness temperature thresholds, such as 207 K [23], 215 K [24], and 235 K [25]. Liu et al. [26] used visible and near-infrared channel data instead to obtain cloud optical thickness and effective radius of cloud droplets, statistically relating them to the probability of precipitation occurrence and achieving a direct test for discriminating precipitation cloud pixels. These traditional methods are mainly based on statistics and establish approximate relationships between the channels of satellite and precipitation clouds. They are easy to use, but their disadvantages are also very obvious. The relationship between cloud top properties and precipitation is very complex and non-linear, but the relationship fitted by these methods is relatively simple [27,28].

With the advent of artificial intelligence, machine learning and deep learning (DL) methods have been used to explore precipitation cloud identification, climate change, natural disaster prediction, and so on [27]. Mecikalski et al. [28] suggested that the IR multi-spectral band difference can be used to identify precipitation clouds before the onset of strong convection based on the observed satellite IR channel brightness temperature difference and weather radar reflectivity data during strong convective weather. Based on the fused spectral and radar observations from tropical rain-measuring satellites during the plum rain period in the Yangtze–Huaihe valley, Fan et al. [29] developed a model to retrieve precipitation intensity based on a random forest algorithm. In this algorithm, the cloud top spectral information of the precipitating cloud is used as input. Moreover, they further explored the relationship between the microphysical characteristics of precipitation clouds and the variation in precipitation intensity during the plum rain period. Hayatbini et al. [30] developed a gradient-based cloud image segmentation algorithm that integrates the magnitude of morphological image gradients to separate the boundary of the cloud system and patch. The algorithm captured the edge objects of different fineness in the remote sensing images to effectively identify precipitation clouds. Kühnlein et al. [31] presented the random forest ensemble classification and regression technique. And multi-spectral visible/IR data from Meteosat Second Generation (MSG) Spinning Enhanced Visible and Infrared Imager (SEVIRI) data are used to improve precipitation estimates during day, night, and nychthemeron. Zheng et al. [32] conducted a study on the automatic identification of strong convective precipitation cloud masses at sea based on Deep Belief Networks using spectral features of Himawari-8 satellite images and texture features extracted based on spectral features. Zhang et al. [33] constructed a Double-Stream Fully Convolutional Network to extract convective clouds from Himawari-8 satellite images. Sadeghi et al. [34] used IR and WV channels (10.7 and 6.7 μm) from the Geostationary Operational Environmental Satellite (GOES) to estimate precipitation based on a convolutional neural network (CNN). Wang et al. [35] developed a novel DL-based algorithm, which proves the effectiveness of the CNN combined with inputs of four physical parameters (CTT, CTH, CP, and WV).

The DL method is more suitable for processing big data with multiple features in practical applications [36], especially for remotely sensed images with high spatiotemporal and multi-spectral resolutions. As far as we know, most of the current precipitation cloud identification is done purely using IR data and only for daytime or nighttime, which cannot meet the requirements of all-weather monitoring. To further investigate the capability of the DL-based algorithm in precipitation cloud identification, this paper proposes a novel end-to-end neural network based on CNN, PCINet, to achieve regional all-weather precipitation cloud identification. PCINet is designed with an encoder–decoder structure. In order to further improve the problem of loss of details in the pooling operation, we propose a skip connection constraint strategy to strengthen the network’s recovery of low-level features. Moreover, we propose a multi-scale structure to enhance the receptive field of the model, so as to take into account precipitation cloud images of different scales. We compared PCINet with previous cloud segmentation methods, and the results show that our proposed algorithm achieves better performance. Due to the FY-4A/AGRI providing images with high spatiotemporal resolution, the proposed method has a good performance in near-real-time all-weather precipitation cloud identification in the study area, while also performing well on long-time series. In addition, once the model is trained, the PCINet has the potential to serve as an algorithm for operational precipitation cloud identification.

The rest of this article is organized as follows. Section 1 introduces the study area and the data used in this article. Section 3 details the methodology and Section 4 introduces the hyperparameter settings of methodology and performance metrics. Section 5 describes the experimental results and discussions. Section 6 is the conclusion.

## 2. Study Area and Data

This section introduces the study area and the collected data for training and evaluating the PCINet, including the FY-4A data and GPM precipitation product data.

### 2.1. Study Area

**Figure 1:**
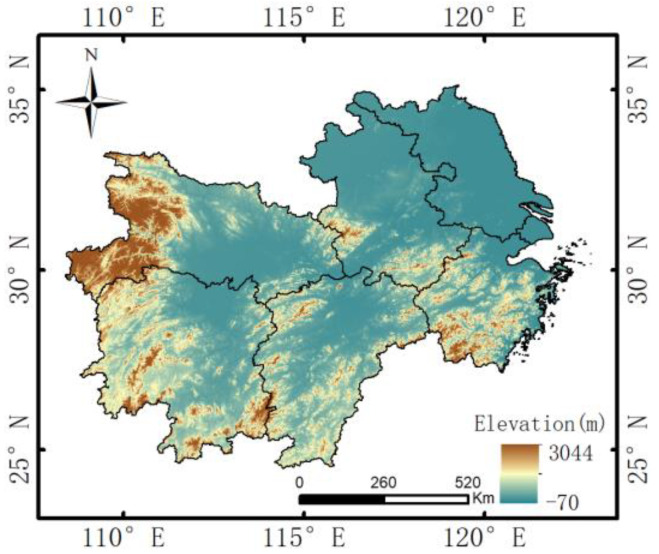
Geographic coverage of the study area.

This work only focuses on a rectangular area in the middle and lower reaches of the Yangtze River, one of the three major plains in China (24–35° N, 108–123° E) (Figure 1). This area has a typical subtropical monsoon climate with mild winters and little rain, and hot, humid summers. According to the research [37], the volume, frequency, and density of summer precipitation in the study area are significantly higher than those in other regions of China. Various training and testing samples for convective precipitation are available to build the precipitation cloud identification model, making the area suitable for the precipitation study. In recent years, frequent and persistent extreme plum rainfall in the middle to lower reaches of the Yangtze River has made the precipitation cloud identification system vital for early warning of hydrological hazards, such as flooding and landslides [37].

### 2.2. FY-4A Data

FY-4A is the first satellite of China’s second-generation geostationary meteorological satellite, which was launched on 11 December 2016 [38]. The Advanced Geosynchronous Radiation Imager (AGRI) onboard the FY-4A satellite (FY-4A/AGRI) has more spectral channels, including visible, short-wave IR, mid-wave IR, and long-wave IR [39]. Moreover, FY-4A/AGRI has the capability of a 15 min full-disk scan and a 5 min scan of the China region to obtain observation images. The visible channel has a high spatial resolution, and its radiation signal reflects the cloud thickness and cloud droplet particle density information, but it is only applicable to daytime. The lower the IR brightness temperature of the precipitation cloud, the higher the precipitation. This relationship can be effectively utilized for precipitation cloud identification, and the IR channel can provide all-weather observation. In this study, we propose to add visible/near-infrared channel information to the precipitation cloud identification in the daytime. Therefore, we considered four physical parameters (CTH, CTT, CP, and WV) to identify precipitation clouds in the nighttime, and we additionally added CWP as input in the daytime. This is because the visible channels have no data at night. The spectral parameters and the corresponding channels are detailed in Table 1.

### 2.3. GPM IMERG Precipitation Product Data

The Global Precipitation Measurement (GPM) mission is co-sponsored by the National Aeronautics and Space Administration (NASA) and Japan Aerospace Exploration Agency (JAXA) in partnership and provides global precipitation products [40]. The GPM satellites are mainly equipped with two types of sensors, the GPM Microwave Imager (GMI) and the Dual-frequency Precipitation Radar (DPR). The former is used to measure precipitation intensity and type, and the latter can penetrate the clouds to observe the internal structure of storms [41]. Therefore, the combination of multi-sensor information makes the precipitation products of GPM more accurate.

The Integrated Multi-satellitE Retrievals for GPM (IMERG) is the GPM Level 3 multi-satellite precipitation algorithm. It not only combines microwave and IR precipitation estimates but also lunar-scale precipitation data from all available satellite constellations to derive global precipitation products [42]. There are three types of products in IMERG, including IMERG-Early (IMERG-E), IMERG-Late (IMERG-L), and IMERG-Final (IMERG-F). IMERG-E and IMERG-L are near-real-time precipitation products, and the IMERG-F represents a post-near-time product combined with the Global Precipitation Climatology Center (GPCC) instrument data. So, it is highly accurate in determining rainy and convective areas, which can be used as the label for training the precipitation cloud identification models [41].

## 3. Materials and Methods

In this study, the precipitation cloud identification task is first converted into an image-to-image translation problem. We propose a multi-scale structure for CNNs based on the encoder–decoder architecture. And a skip connection constraint strategy is presented in this section. The main reasons are as follows. (1) Different precipitation types have various rainfall coverage and the multi-scale structure of the algorithm can enlarge the receptive field of the network to take into account precipitation with different scales; (2) the skip connection between low-resolution feature layers and high-resolution feature layers can reduce information damage due to pooling operations; (3) the boundaries of precipitation cloud clusters and non-precipitation cloud clusters are fuzzy, and are even included in the non-precipitation cloud clusters. The skip connection constraint strategy can be used to strengthen the low-level feature extraction.

### 3.1. Overview of Architecture

**Figure 2:**
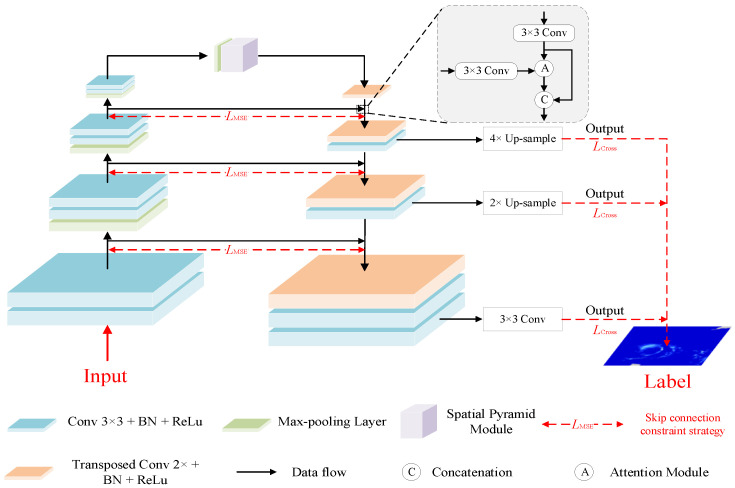
Detailed architecture of the PCINet model. The input of the PCINet is the catenated imagery according to Table 1, including the IR_13.5_, IR_10.7_, WV_6.25_, ΔT_6.25–10.7_, ΔT_10.7–13.5_, and the bands of CWP. The input bands of PCINet need to meet the requirements of daytime, nighttime, and nychthemeron in Table 1.

Figure 2 shows the detailed architecture of the proposed PCINet model. The model’s input consists of five physical parameters, including the CTH, CTT, CP, WV, and CWP. These parameters are replaced by the bands in Table 1, and the input is to be determined according to the time of these images. The output is the prediction in the form of a single-channel binary precipitation cloud identification image. The architecture of the PCINet is based on the structure of the encoder–decoder. On the left side of the Spatial Pyramid Module (SPM) is the encoder, which reduces the image size and extracts low-level features through the convolutional layer and max-pooling. And on the right side of the SPM is the decoder, which extracts the high-level features of the precipitation cloud and recovers the output results’ resolution by a transposed convolutional layer. In the PCINet, the kernel size and stride of convolutional layers are 3 × 3 and 1, respectively. And the kernel size and stride of transposed convolutional layers are 2 × 2 and 2, respectively. The batch normalization (BN) layers are included after the convolutional layer and transposed convolutional layer in the encoder and decoder, and the activation function used in PCINet is rectified linear unit (ReLU). In the encoder, the number of filters for the CNN is set to 64, 128, 256, and 512. And the filters in the decoder are the same as those in the encoder, which was determined by the skip connection. In the middle part of the model, we introduce a spatial pyramid module for multi-scale feature extraction, which was detailed in Section 3.2. Moreover, we creatively design the skip connection constraint strategy (indicated by the red double-headed arrow in Figure 2) for PCINet, which is a penalty for the feature map between the encoder and decoder. In addition, we enhance the ability to identify precipitation clouds by combining the feature maps in the encoder with the upsampling output through an attention mechanism module. Finally, the output of the network adopts a multi-path strategy, that is, each block in the decoder must output a result using the sigmoid function (Equation (1)). And their results are fed into loss functions by the cross entropy (*L*_Cross_). In Figure 2, the 2× upsample is operated by a transposed convolutional layer, and the parameters are the same as those in the decoder, while the 4× upsample operation is composed of two transposed convolutional layers.

(1) $$Sigmoid\left(x\right)=\frac{1}{e^{-x}}$$

### 3.2. Spatial Pyramid Module

**Figure 3:**
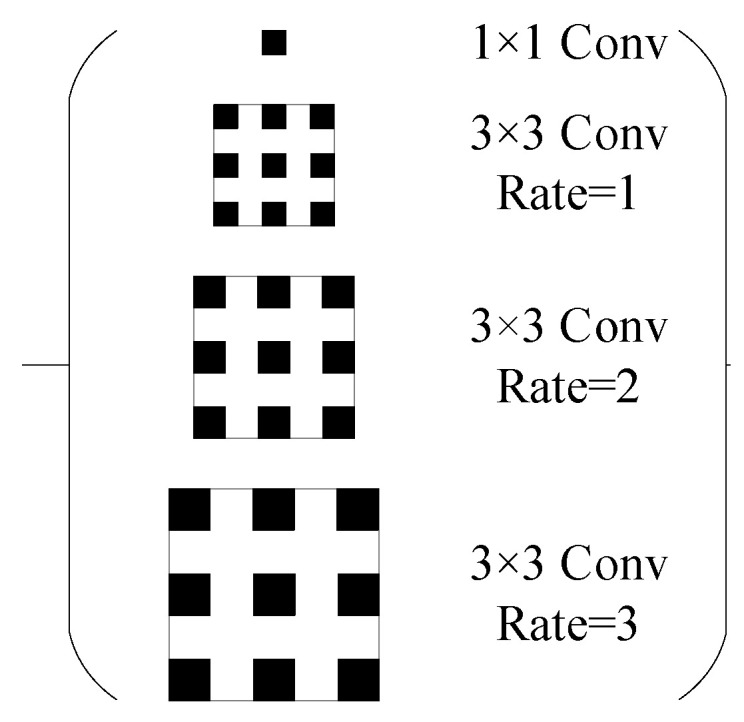
Schematic diagram of the spatial pyramid structure.

We design a spatial pyramid module inspired by the Atrous Spatial Pyramid Pooling (ASPP) module [43] to extract high-level features in the middle layer of the model from multiple scales and use a hybrid dilation convolution with the rate set to a sawtooth shape (Figure 3) of rate 1, 2, 3 to extract the feature information of the precipitation cloud from different scales to ensure that the perceptual field can cover the whole region. The size of the precipitation area is usually not regular. In the same scene image, there may be precipitation areas of different sizes, and even scattered precipitation may appear. As shown in Figure 3, 1 × 1 Conv represents the kernel size, and the stride of this convolutional layer is 1 × 1 and 1; everything else is the same. Here, we keep the kernel size and rate of the convolution operation different, but the size of the feature maps is the same, and these maps are concatenated. Previous ASPP modules use the atrous rate of [6,12,18] for multi-scale feature extraction, which is not suitable for the precipitation cloud identification task in this paper. The excessive atrous rate tends to cause the loss of small target object information. The benefits of this modification are demonstrated in the subsequent model comparison experiments.

### 3.3. Attention Mechanism Module

**Figure 4:**
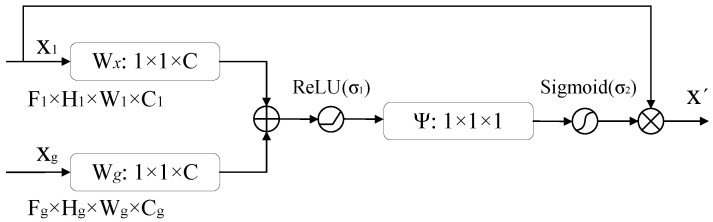
Schematic diagram of the attention mechanism module.

Figure 4 shows the attention mechanism module [44] added to the model allowing the model to focus more on the feature information in the precipitation cloud region. x_l_ (the output of the encoder) and x_g_ (the output of upsampling) are both fed into a 1 × 1 convolution layer that changes them to the same number of channels without changing the size and subsequently accumulates and passes a ReLU activation function. Then, another 1 × 1 convolution layer and a sigmoid function are passed to obtain an importance score from 0 to 1, assigned to each part of the feature map. Finally, this attention graph is multiplied by the x_l_ input to produce the final output of this attention mechanism module.

### 3.4. Loss Function

In order to optimize the parameters in the precipitation cloud identification model, we use the mixed loss as the loss function of the model, defined as follows:

(2) $$L_{\mathrm{mix}\;}=\sum_{i=1}^{M}L_{\mathrm{cross},i}+\lambda \sum_{j=1}^{N}L_{\mathrm{mse},j}$$

where *M* is the number of upsampling layers, $L_{cross,i}$ is the cross-entropy loss [45] at upsampling layer *i*, and λ is the importance of the loss term. *N* is the number of hidden layers, and $L_{mse,j}$ is the MSE of the encoder and decoder at layer *j*.

(3) $$L_{MSE}=\frac{1}{N}\sum_{i=1}^{N}{\left||y_{i}-{y_{i}}^{\prime }|\right|}_{2}^{2}$$

where *y_i_* and *y_i_*′ represent the real and the estimated precipitation amounts, respectively. *N* is the total number of pixels.

As shown in Figure 2, we use bilinear interpolation to recover the output after each upsampling operation to ensure the same size as the labels and then compute the cross-entropy loss between the prediction and labels.

### 3.5. Baseline Models

PCINet is based on the architecture of Unet [46], which is composed of encoder, decoder, and skip connections. Unlike Unet, the skip connection constraint strategy is proposed in PCINet, and a multi-output structure is present for it. The motivation for the skip connection constraint strategy is to settle the problem of information loss caused by pooling and upsampling operations in the encoder and decoder. The multi-output structure is used to strengthen the recovery of features in the middle layers. Therefore, in order to evaluate the effectiveness of PCINet, the Unet is utilized as a benchmark for comparison. In addition, FCN-8s [47], PSPnet [43], Deeplab V3+ [48], and SegNet [49] are also used for comparison. In the research of [47], a fully convolutional network (FCN) is proposed, and it is trained end-to-end, pixel-to-pixel on semantic segmentation. Unlike Unet, there are fewer feature maps in the upsampling path of FCN-8s. In the study of [43], a Pyramid Scene Parsing Network (PSPnet) is designed based on the FCN. In PSPnet, the pyramid pooling module is proposed as the effective global context prior. Unlike the spatial pyramid module in Figure 2, the pyramid pooling module processes the feature maps from the last layer by pooling operation. DeepLab V3+ is based on fully convolutional networks and uses the atrous convolution instead of the convolution operation in FCN. SegNet is proposed in [49] and is also constructed based on the framework of the encoder–decoder. Unlike the Unet and PCINet, instead of a skip connection, this model uses pooling indices when recovering the information after the upsampling operation, which is more conducive to saving memory.

## 4. Experiment Setup

### 4.1. Data Preprocessing

The data of FY-4A and IMERG require consistent spatiotemporal resolution before the experiment. Since the data we use are all calibrated products, we only need to align their spatiotemporal resolution when using them. We re-grid the IMERG-F precipitation data onto the FY-4A grid using a bilinear interpolation method because the spatial resolution of the FY-4A satellite data is 4 km (0.04°), while the spatial resolution of the IMERG-F precipitation data is 0.1° (around 10 km). The second task is temporal resolution matching. The temporal resolution of the FY-4A and IMERG-F data is about 5 and 30 min, respectively. To make them consistent, we only select FY-4A satellite images with the same recording time as IMERG-F precipitation data. In [50], 0.5 mm/h is taken as the threshold for precipitation identification, but in this paper, we make precipitation cloud identification labels based on the IMERG-F precipitation data with a threshold of 0.2 mm/h.

Considering the specificity of the precipitation situation in the study area, June to September 2019–2020 are chosen to obtain sufficient precipitation and convective-type samples. The samples are randomly divided as the data for training and validation sets. Samples from June to September 2021 are selected as an independent test set to verify the model’s capability in identifying precipitation clouds. In order to reduce the impact of the imbalance between daytime and nighttime data on the experimental results, we involve as much data as possible in the model training.

### 4.2. Hyperparameter Setting

The central processing unit (CPU) and graphics processing unit (GPU) used in this research are Intel(R) Core(TM) i5-7500 CPU and NVIDIA GTX 1080, respectively. And the architectures of the models used in this research are based on Pytorch. The initialization weights of the models are drawn from a normal distribution with a mean of 0 and a standard deviation of 1. The parameters are optimized by the RMSProp optimizer [51] and the initial learning rate is set to 1 × 10^−4^. After every 50 epochs, the learning rate is adjusted to one-tenth of the previous rate. The batch size of PCINet is set to 4.

### 4.3. Evaluation Metrics

Two types of metrics are used to evaluate the performance of precipitation cloud identification. The first group metric is to evaluate the performance of image segmentation, including mean intersection over union (M), accuracy (A), precision (P), recall (R), f-score (F), and error rate (ER). And the second set of metrics is used to evaluate whether the precipitation pixels are misclassified or missed, including the probability of detection (POD), false alarm ratio (FAR), and critical success index (CSI).

## 5. Results and Discussions

To verify the effectiveness of the PCINet model, we compare the precipitation cloud identification of PCINet with five other deep learning models (Unet, PSPNet, DeeplabV3+, SegNet, and FCN-8s) from both qualitative and quantitative perspectives over the test periods. We evaluate PCINet qualitatively by visualizing the results of two events during the daytime and nighttime and evaluate the pixel-level identification accuracy of long-time series. In terms of quantitative evaluation, we use evaluation metrics to analyze the performance gain or loss of the PCINet model compared to other baseline models, as well as the improvement of daytime precipitation cloud identification accuracy with the addition of visible/near-infrared feature information.

### 5.1. Comparison of Image Segmentation Performance

Table 2 summarizes the overall image segmentation performance of PCINet and the five baseline models during the test period (June–September 2021) for daytime, nighttime, and nychthemeron. The PCINet model reflects the best performance for the three time divisions, which indicates that PCINet is more refined for precipitation cloud segmentation. Among the five baseline models, Unet performs the best. As shown in the framework of Figure 2, more location and detailed information are included in the low-level feature maps because of their higher resolution. With the deepening of the convolutional layers, high-level feature maps are obtained. Although these feature maps have stronger semantic information, their resolution is greatly reduced due to the pooling operation. Thus, more high-resolution detailed information contained in the high-level feature maps can be retained by fusing low- and high-level feature maps through the skip connections. Compared with Unet, PCINet, which is also based on the encoder–decoder model, increases M, A, P, R, and F by 5.5, 2, 3.8, 5.1, and 4.6%, respectively. All models perform better in identifying daytime than nighttime precipitation clouds. The DeeplabV3+ model, which shows the most improvement, improves M, A, R, and F by 7.8, 1.2, 9.1, and 8%, respectively. SegNet has the largest improvement in P, with a 4% improvement. This proves that increasing the information of the 0.6, 0.8, and 1.6 μm channels can help to improve the accuracy of cloud phase state identification for thin cirrus clouds in the daytime. The visible channel is a non-water absorption channel, and its radiation signal reflects the information on cloud thickness and cloud droplet particle density. Thus, the visible channel signal is also an important indication of precipitation cloud identification. Identifying precipitation clouds from single-channel information is more limited. Therefore, it is more effective to identify precipitation clouds by pooling the advantages of each channel. In terms of overall promising performance, PCINet achieves the best performance within these precipitation cloud identification algorithms. These results show that the multi-scale structure and skip connection constraint strategy proposed in this article are effective. Finally, comparing the results of PCINet in daytime, nighttime, and nychthemeron, it can be seen that the values of these six metrics of nychthemeron are close to those in nighttime but higher than it. This may be due to the addition of daytime training samples compared with those in nighttime, which improves the results in nychthemeron. However, the results of daytime are higher, because visible band data can be used during the daytime, while only infrared data can be used for nighttime and nychthemeron. This means that the visible band is effective in improving the identification of precipitation clouds.

### 5.2. Comparison of Daytime Precipitation Cloud Identification

Table 3 summarizes the overall precipitation cloud identification performance of PCINet and the five baseline models during the daytime of the test period. The PCINet model shows the best performance in the daytime. It increases POD and CSI by 1.4 and 2.3%, respectively, and decreases FAR by 1.5%, compared to the Unet model, which is the best-performing of the five baseline models. It can be concluded that adding the attention mechanism module between the model encoding and decoding layer can restore the lost information during the execution of the upsampling layer and improve the model’s attention to precipitation cloud information. The performance gain means that the PCINet model can identify more precipitation clouds in the daytime and has fewer false positives for non-precipitation cloud regions.

**Figure 5:**
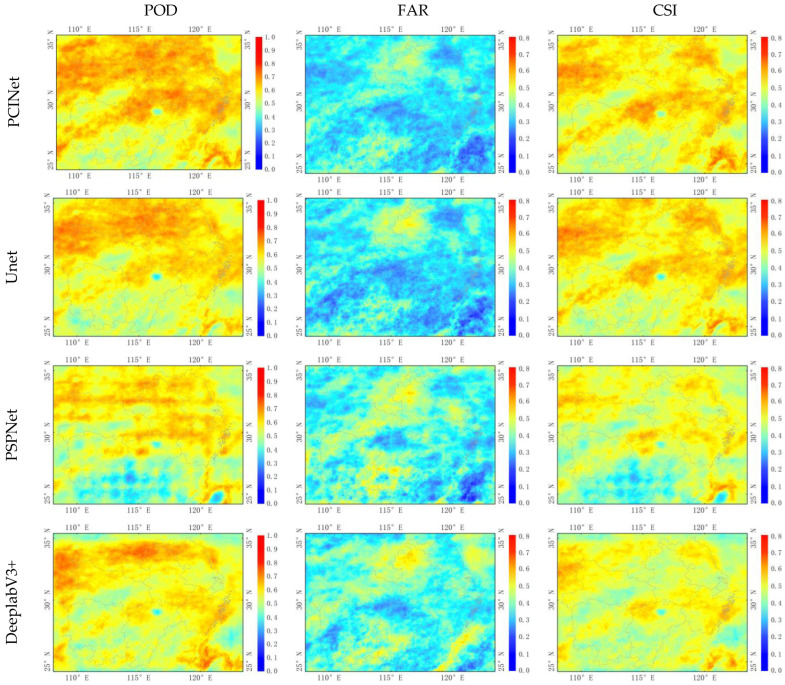

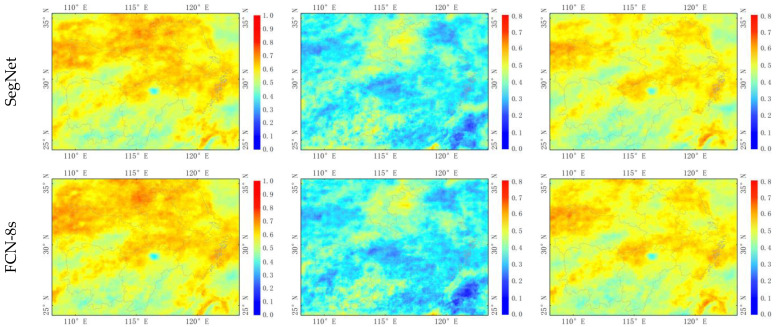
Spatial distribution of the evaluation metrics (POD—the first column, FAR—the second column, and CSI—the third column) for PCINet (the first row), Unet (the second row), PSPNet (the third row), DeeplabV3+ (the forth row), SegNet (the fifth row), and FCN-8s (the sixth row) in the daytime over the test period (June–September 2021).

Figure 5 shows the spatial distribution of PCINet and baseline models’ precipitation cloud identification metrics (POD, FAR, CSI) in the daytime throughout the test period (June–September 2021). The warm colors indicate high measurement values and cold is the opposite. For POD and CSI, the expected value is 1. For FAR, the desired value is 0. All evaluation metrics are calculated for each pixel and at a half-hourly time scale over the study area for the entire test period. For POD, PCINet has a significant improvement compared to the five baseline models. In the central region (28–31° N, 113–118° E), PCINet performs better and identifies more precipitation clouds. However, the overall spatial distribution of POD for PSPNet appears to be less homogeneous, especially in the southern region, where large areas of precipitation cloud are not identified. For FAR, the PCINet and Unet models have almost similar performance and even perform better in the southern region than the other four baseline models, which is consistent with the performance of FAR in Table 3 (PCINet is 0.246 and Unet is 0.231). The PSPNet and DeeplabV3+ perform the worst. As mentioned earlier, PSPNet and DeeplabV3+ use a large atrous rate. Thus, increasing the model field also leads to the loss of precipitation cloud information. For CSI, both the PCINet and Unet models show significant improvements in the northern region, PSPNet scores lower in the southern region, consistent with the lowest CSI values in Table 3, and SegNet and FCN-8s display average performances. The results show that, overall, the PCINet model can identify precipitation clouds in the daytime more accurately.

**Figure 6:**
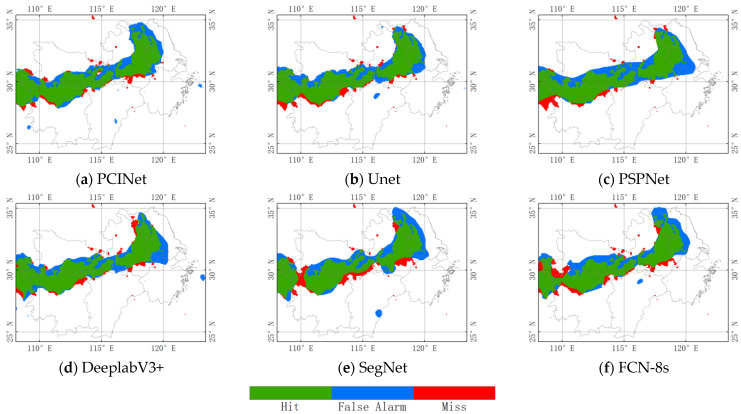
Spatial distribution of precipitation cloud identification at 00:30 (UTC) on 17 July 2021. The green, blue, and red colors represent hit, false detection, and miss, respectively. The white color indicates areas with no precipitation cloud during the corresponding period.

In order to verify the performance of the PCINet model on practical precipitation events during daytime in the study area, we selected the precipitation event that occurred on 17 July 2021 for testing. From 0:00 a.m. to 0:30 a.m., a large amount of precipitation was observed in the north-central region of the middle and lower reaches of the Yangtze River. Figure 6 shows the precipitation cloud identification results from PCINet and the five baseline models. It is obvious that PCINet captures the most precipitation cloud pixels, but there are also small precipitation cloud regions that are incorrectly detected. Unet misses some precipitation cloud pixels in the southern regions, and PSPNet and DeeplabV3+ incorrectly detect many no-precipitation cloud regions. SegNet and FCN-8s perform the worst in the precipitation cloud identification task during this daytime and miss most precipitation cloud pixels in the area (28–30° N, 109–111° E). The results show that the PCINet is more effective and accurate in capturing the precipitation cloud, which means that the PCINet model can extract more useful precipitation-related features from satellite data than the other models.

### 5.3. Comparison of Nighttime Precipitation Cloud Identification

Table 4 exhibits the performance of PCINet and the five baseline models during nighttime in the test period. For POD and FAR, PCINet performs the best while DeeplabV3+ performs the worst, which means that PCINet tends to identify precipitation cloud regions accurately, while DeeplabV3+ tends to over-identify non-precipitation areas as precipitation cloud areas. PSPNet performs slightly better than DeeplabV3+. SegNet and FCN-8s are relatively balanced, and their performances in identifying precipitation cloud regions are almost identical (POD of 0.533 and 0.537, respectively). However, FAR of FCN-8s is lower than that of SegNet, indicating that FCN-8s has higher precision. PCINet has the highest CSI compared to the other models. We also compare the metrics of each model in Table 4 with those in Table 3. POD and CSI of DeeplabV3+ improve the most, with an increase of 19.2 and 14.8%, respectively, while FAR of SegNet has the least improvement (decrease of 6.1%). The overall result shows that each model performs better in the daytime than nighttime precipitation cloud identification, effectively demonstrating that adding visible/near-infrared spectral information to the daytime precipitation cloud identification model improves the results of PCINet.

**Figure 7:**
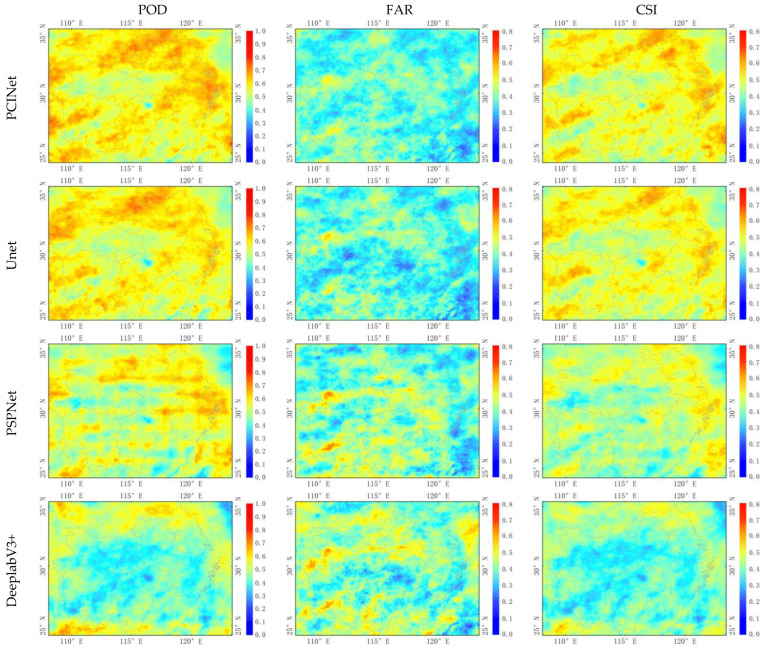

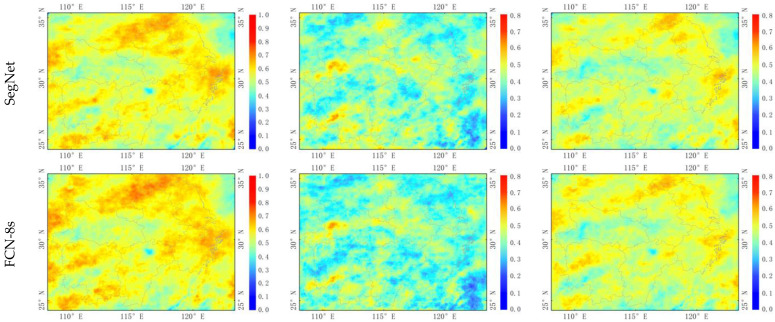
Spatial distribution of the evaluation metrics (POD—the first column, FAR—the second column, and CSI—the third column) for PCINet (the first row), Unet (the second row), PSPNet (the third row), DeeplabV3+ (the fourth row), SegNet (the fifth row), and FCN-8s (the sixth row) in the nighttime over the test period (June–September 2021).

Figure 7 shows the spatial distribution of precipitation cloud identification metrics (POD, FAR, CSI) of PCINet and the baseline models in the nighttime throughout the test period (June–September 2021). Compared to the Unet, PSPNet, Deeplab V3+, SegNet, and FCN-8s, the PCINet model shows a higher POD value, while PSPNet performs the worst, which is consistent with the results in Table 4. The average FAR of DeeplabV3+ is almost the same as that of PSPNet and much higher than the other models. For FAR, more cold color pixels for PCINet and Unet indicate that both models rarely identify cloud-covered areas as precipitation cloud areas. For CSI, more warm color pixels show that the PCINet model outperforms the baseline models. Overall, the PCINet model shows a more balanced and better performance in nighttime precipitation cloud identification.

**Figure 8:**
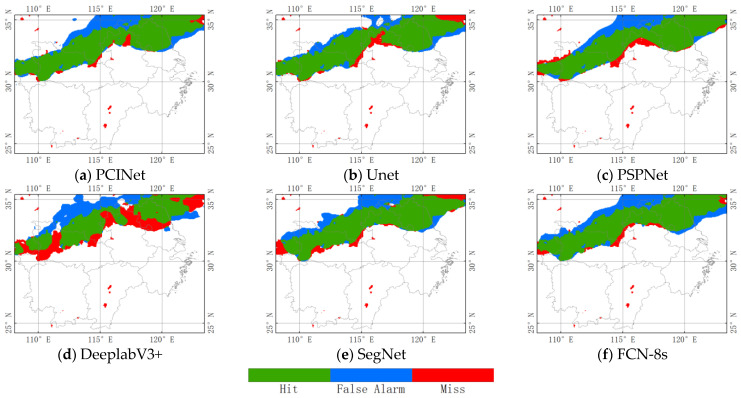
Spatial distribution of precipitation cloud identification at 21:30 (UTC) on 6 July 2021. The green, blue, and red colors represent hit, false detection, and miss, respectively. The white color indicates areas with no precipitation cloud during the corresponding period.

Figure 8 shows the precipitation cloud identification result of PCINet and the five baseline models at 2130 UTC on 6 July 2021. PCINet can correctly identify almost all precipitation cloud areas, and the number of false detection pixels is relatively low among the six DL-based models. Unet can reduce the false detection regions, but it also misses some precipitation cloud pixels. PSPNet not only misses a large number of precipitation cloud pixels but also incorrectly detects many of them. DeeplabV3+ misses the most precipitation cloud pixels and fails to accurately delineate the areas. SegNet and FCN-8s have average performances, and the edge of the precipitation cloud is very blurred. The results once again show that the PCINet model is more effective and accurate in depicting precipitation cloud regions during this precipitation cloud event compared to the baseline deep learning model.

## 6. Conclusions

In this article, we propose a precipitation cloud identification model based on an encoder–decoder structure. In the middle layer, a multi-scale structure based on ASPP is used to extract precipitation cloud information in multiple resolutions. Moreover, a skip connection constraint strategy is proposed to strengthen the capability of the network in the recovery of lost information when layers go deeper. To validate the performance of PCINet, we compare it with the results identified by the baseline models. The experiment results show that PCINet outperforms the other models whether it is daytime, nighttime, and nychthemeron. Moreover, we compared the results of PCINet in these three time periods. Their results show that the performance metrics of the nychthemeron are close to those of nighttime but slightly higher than them. And the results of daytime are superior to the other two. This means that the visible band of the satellite can provide more information about precipitation for the DL-based precipitation cloud identification algorithm. In addition, we evaluate the spatial performance of the different models during two heavy precipitation events. PCINet has a more consistent and accurate spatial distribution. Model evaluation during the test period shows that the proposed model has a more homogeneous and consistent performance for the various evaluation metrics. The overall performance show that this algorithm improves the cloud identification performance, and the combination of low- and high-level features in the decoder can give a richer position, edge, and detailed information. In the future, we plan to refine the classification of clouds according to precipitation types and take geographic information and other meteorological elements into consideration to better learn the precipitation region features.

## Figures and Tables

**Table 1:** Spectral parameters used in models.

Types	Daytime	Nighttime	Nychthemeron
CTH	IR_13.5_	IR_13.5_	IR_13.5_
	ΔT_6.25–10.7_	ΔT_6.25–10.7_	ΔT_6.25–10.7_
CTT	IR_10.7_	IR_10.7_	IR_10.7_
CP	ΔT_10.7–13.5_	ΔT_10.7–13.5_	ΔT_10.7–13.5_
WV	WV_6.25_	WV_6.25_	WV_6.25_
CWP	VIS_0.6_		
	VIS_0.8_		
	NIR_1.6_		

The CTH, CTT, CP, WV, and CWP are the physical parameters used in this work to identify precipitation clouds, which can be approximated by the spectral data of the satellite. In Table 1, ΔT means the difference between the two bands and the subscript represents the center wavelength of the band. Since the visible band only has data in the daytime, the CWP is not used in nighttime and nychthemeron.

**Table 2:** Summary of image segmentation performances over the test periods.

	Model	M	A	P	R	F	ER
Daytime	PCINet	0.748	0.919	0.865	0.830	0.843	0.081
	Unet	0.711	0.904	0.846	0.798	0.813	0.096
	PSPNet	0.666	0.886	0.816	0.759	0.774	0.114
	DeeplabV3+	0.674	0.888	0.817	0.772	0.785	0.112
	SegNet	0.684	0.893	0.826	0.775	0.792	0.107
	FCN-8s	0.691	0.896	0.831	0.782	0.797	0.104
Nighttime	PCINet	0.726	0.915	0.849	0.810	0.825	0.085
	Unet	0.674	0.899	0.826	0.759	0.779	0.101
	PSPNet	0.637	0.885	0.796	0.728	0.744	0.115
	DeeplabV3+	0.596	0.874	0.779	0.681	0.705	0.126
	SegNet	0.648	0.887	0.786	0.741	0.757	0.113
	FCN-8s	0.655	0.891	0.807	0.745	0.763	0.109
Nychthemeron	PCINet	0.742	0.916	0.859	0.825	0.838	0.084
	Unet	0.687	0.896	0.821	0.783	0.794	0.104
	PSPNet	0.639	0.880	0.798	0.734	0.749	0.120
	DeeplabV3+	0.597	0.870	0.792	0.681	0.708	0.130
	SegNet	0.659	0.887	0.805	0.755	0.769	0.113
	FCN-8s	0.667	0.889	0.811	0.762	0.776	0.111

**Table 3:** Daytime precipitation cloud identification performance over the test periods.

	Model	POD	FAR	CSI
Daytime	PCINet	0.651	0.246	0.554
	Unet	0.637	0.231	0.531
	PSPNet	0.564	0.277	0.459
	DeeplabV3+	0.594	0.279	0.476
	SegNet	0.597	0.264	0.489
	FCN-8s	0.608	0.256	0.499

**Table 4:** Summary of nighttime precipitation cloud identification performance over the test periods.

	Model	POD	FAR	CSI
Nighttime	PCINet	0.583	0.283	0.481
	Unet	0.558	0.269	0.459
	PSPNet	0.500	0.318	0.399
	DeeplabV3+	0.402	0.338	0.328
	SegNet	0.533	0.325	0.421
	FCN-8s	0.537	0.303	0.431

## Data Availability

Not applicable.
